# Psychosocial Well-Being, Mental Health, and Available Supports in an Arab Enclave: Exploring Outcomes for Foreign-Born and U.S.-Born Adolescents

**DOI:** 10.3389/fpsyt.2021.632031

**Published:** 2021-04-08

**Authors:** Ilana Seff, Alli Gillespie, Cyril Bennouna, Wafa Hassan, Mackenzie V. Robinson, Michael Wessells, Carine Allaf, Lindsay Stark

**Affiliations:** ^1^Mailman School of Public Health, Columbia University, New York City, NY, United States; ^2^Brown School at Washington University in St. Louis, St. Louis, MO, United States; ^3^Department of Political Science, Brown University, Providence, RI, United States; ^4^Global Educational Excellence, Ann Arbor, MI, United States; ^5^Qatar Foundation International, Washington, DC, United States

**Keywords:** adolescents, refugees, mental health, MENA, immigrant paradox, gender, acculturation, ethnic enclaves

## Abstract

**Introduction:** Few studies have assessed the impact of displacement, resettlement, and discrimination on well-being outcomes for adolescent refugees resettled within the U.S. Conducted in three charter schools in the intergenerational Arab enclave of the Detroit Metropolitan Area, this mixed-methods study assessed the mental health and psychosocial support for both U.S.- and foreign-born adolescents from the Middle East and North Africa region.

**Methods:** A quantitative survey was used to collect data on 176 students. Key outcomes included hope, prosocial behaviors, resilience, depressive, anxiety, externalizing symptoms, stressful life events, perceived social support, and sense of school belonging. Differences in outcomes between U.S.- and foreign-born students were compared using *T*-tests. Regression analysis explored whether outcomes were gendered and correlated with years in the U.S. for foreign-born students. Qualitative data collection included key informant interviews with school staff and community service providers, student focus group discussions, and caregiver interviews. Interview transcripts were analyzed using thematic analysis and the constant comparative method.

**Results:** No statistically significant differences between the foreign-born and U.S.-born groups were observed. However, analysis revealed that resilience decreased for male students with time spent in the U.S. Qualitative themes illuminated these results; shared cultural heritage allowed newcomer students to access relevant language and psychosocial support, while inter- and intra-group peer relationships strengthened students' dual language skills and identity formation. However, shifting gender expectations and role hierarchies for newcomer students revealed boys' increased stressors in the family domain and girls' better accessed support in the school context.

**Conclusion:** The existence of an immigrant paradox in this enclave setting was not supported. Instead, findings highlight the reciprocal value of peer-based mentorships and friendships between U.S.- and foreign-born students with similar cultural backgrounds, the importance of social and emotional curricula and cultural competency training within schools, and the gendered effects of acculturation.

## Introduction

An unprecedented 70 million forcibly displaced people (1, 2), rising Islamophobia, and anti-immigration sentiment make this an important moment to ask how to support the mental health and psychosocial well-being of refugees, including Arab refugees who have resettled in the U. S. from Syria, Iraq, and the wider Middle East and North Africa (MENA) region. A growing body of evidence has documented that Arab immigrant and refugee populations resettled in the U.S. face significant discrimination and worsened mental health (3). Further, a comparison of Arab refugees and Arab non-refugees in the Detroit Metropolitan Area (DMA) showed that adult refugees exhibit higher levels of depression and anxiety than non-refugees (4).

Few studies, however, have focused on adolescent populations from the MENA region who resettle in the U.S. These adolescents must process their loss of home and experiences of migration while navigating a new cultural environment and educational system during a formative developmental period (5). As adverse exposures during adolescence can have profound and lasting effects, mitigating mental health challenges for adolescent refugees is critical for ensuring lifelong well-being (6, 7). Potentially offsetting these risks is the “immigrant paradox,” a pattern whereby newer immigrants exhibit healthier outcomes than their U.S.-born peers (4, 8, 9). Yet it cannot be assumed that Arabic speaking adolescent refugees will benefit from this paradox.

Schools are particularly well-positioned to support newcomer adolescents beyond their family and community networks. Refugees who have a strong sense of belonging in their schools tend to exhibit lower levels of psychosocial distress (10–12). Further, schools may provide newcomers an accessible, familiar, and non-stigmatizing venue for obtaining various mental health services and psychosocial supports (13, 14). Such supports may range from social and emotional learning curricula to more specialized services, such as cognitive behavioral therapy. However, schools often lack the financial and human resources capacity and evidence-based guidance to achieve well-integrated, culturally relevant, and responsive support systems (15, 16).

The adaptation challenges faced by adolescent refugees from the MENA region are not well-documented. This study aims to fill this gap by assessing the mental health and psychosocial well-being needs, resilience, and existing support mechanisms of both U.S.- and foreign-born adolescents whose families immigrated to Michigan from conflict-affected MENA countries. It is hoped that the results will inform the development of more effective interventions that support this population.

## Methods

### Study Setting and Participants

Data were collected as part of the Study of Adolescent Lives after Migration to America (SALaMA), a multi-city, mixed-methods study conducted to assess the mental health and psychosocial needs of adolescent students from the MENA region (17). The present analysis looked specifically at data collected in Michigan's DMA. For many years, the DMA has been a highly preferred resettlement area for MENA immigrants and forced migrants. In Dearborn, a city in the DMA, nearly 50% of the population identifies as Arab (18). As such, the DMA now serves as one of the largest ethnic and cultural enclaves for Arab immigrants and Arab-Americans (19).

This study took place within three high schools within a charter school system in the DMA. This charter school system is open to all students but was designed to support English language learners, at-risk students, and refugees. Arabic was required as a foreign language course in all three study schools. Given this feature of the study schools, the vast majority of enrolled students were either born in the MENA region or had at least one parent who was born in the MENA region.

### Quantitative Study Procedures

Researchers visited all Arabic classes held at each school to invite students to participate in the survey questionnaire. Parental consent forms in Arabic and English were disseminated ~6–8 weeks prior to data collection, and students returned the signed parental consent forms to their Arabic teachers. Quantitative data collection took place during Arabic classes over the course of one day at each school in September and November 2019. After all students in a class block were checked in, the researcher began the informed assent process by explaining the research purpose, procedures, potential risks, and safeguards. Participant confidentiality and follow-up procedures were also discussed. The researcher emphasized that participation was completely voluntary and that not participating would result in no penalty of any kind. The research team also worked with the school system, following survey administration, to access attendance records. Assent for participation in the survey and accessing student records was obtained separately, so students could opt to participate in the survey but not release their records.

Assenting students received a unique study code, which they entered at the beginning of their survey. The self-administered survey took 15–30 minutes to complete online on school-issued devices. Arabic or English versions of the survey were available and included questions on basic demographics and outcomes related to mental health and psychosocial well-being. Attendance records, in de-identified formats, were later linked with the above survey data.

### Quantitative Measures

In order to ensure data validity and comparability with previous and future studies, the survey incorporated items from several instruments that had previously been used with refugee populations in high-income countries. Our analysis examined seven key outcomes of interest related to mental health and psychosocial well-being. Further details on these outcomes and internal consistency can be found in Table 1. Attendance, obtained from school records, was measured by dividing the number of classes for which a student was tardy or absent, respectively, by the number of classes each student attended per day.

**Table 1:** Outcomes of interest.

**Instrument**	**Rationale/Outcome measured**	**Components used**	**Values**	**Internal consistency (Cronbach's alpha)**
Children's hope Scale	Students' hope, as related to agency and pathways for meeting goals	6 items	Average score on all six questions that may take a value from 1 to 6, higher scores reflect greater hope	0.81
Strengths and difficulties questionnaire (SDQ)	Used to screen for poor dimensions of mental health	Two of five sub-scales: Peer Problems and Prosocial sub-scales	Each sub-scale may take value from 0 to 10, higher scores reflect more positive peer relationships or pro-social behaviors	Peer problems: 0.46 (not used) Prosocial behavior: 0.66
Child and youth resilience measure (CYRM)	Resilience among children and adolescents while allowing for cultural variation across multiple settings; previously validated with adolescents in refugee/asylum-seeking contexts	12-item version of the original 28-item scale	12–36, higher values reflecting greater resilience	0.73
Hopkins symptom checklist	Multiple dimensions of mental health, including depressive symptoms, symptoms of anxiety, and externalizing symptoms; previously validated with refugee/asylum-seeking adolescents in multiple languages	37-item version of the original 58-item checklist	All sub-scales may take a value from 1 to 4, higher scores reflect greater symptomology	Depressive symptoms: 0.91 Anxiety symptoms: 0.74 Externalizing symptoms: 0.69
Stressful life events checklist	Exposure to eleven stressful lifetime events and drastic changes in the family in the last year	12 items	0–12, higher values reflecting exposure to a greater number of events	0.69
Multidimensional scale of perceived social support (MSPSS)	Perceived social support from three sources: friends, family, and a significant other; validated with multiple adolescent populations, including Arab American adolescents	12 items	0–7, higher values reflecting greater perceived social support	0.94
Psychological sense of school membership (PSSM) scale	Students' sense of belonging in their school environment; used and validated with adolescent refugee/asylum-seeking populations in the U.S.	18 items	18–90, higher values reflecting greater sense of school belonging	0.83

Source: (17)

### Qualitative Study Procedures

Qualitative research activities were conducted from August through November 2019 and included key informant interviews (KIIs), focus group discussions (FGDs), and caregiver interviews. The research team worked closely with school officials to identify students born—or whose parents were born—in the MENA region to participate in FGDs; caregivers of such students were recruited for interviews. With the assistance of staff members at each school, the team contacted eligible families to explain the purpose of the study and to assess each family's interest in participating and obtain written informed consent. Student assent was also obtained.

The team conducted a total of eight FGDs, which were stratified by gender. Of the four FGDs with girls, one was comprised of entirely newcomer students while one included all U.S.-born students. One of the four FGDs with boys was also entirely newcomer students. The remaining groups included a combination of both newcomer and U.S.-born students. Ultimately, 39 students and 12 caregivers assented/consented to participate in the qualitative component of the study. While all FGD students' caregivers were invited to participate, those who accepted were mostly women.

Key informants were selected in consultation with school officials and local organizations, with a focus on recruiting participants who provide services for refugee students and their families. The 13 key informants who agreed to participate in the study included school administrators, teachers, guidance counselors, therapists, case workers, and spiritual leaders from the community. Both caregiver interviews and KIIs were semi-structured and took place in local schools or at the key informant's place of work. One or two researchers were present during interviews. Arabic interpreters were provided for interviews as requested. Participants were asked about their job responsibilities and invited to discuss newcomer students' challenges and strengths related to school performance, social and emotional learning, language proficiency, and relationships. Questions were largely tailored to participants' experiences and roles working with refugee youth.

The FGD questions focused on student experiences, including their relationships, their families, and the school system. Additionally, students completed a participatory ranking activity at the end of each FGD (20). During this exercise, participants collectively brainstormed and ranked ideas and recommendations for improving newcomer students' experiences at their school.

Caregiver interviews assessed family experiences adjusting to life in the U.S. Caregivers were asked about their displacement and resettlement, as well as their children's experiences in their new school. Caregivers also discussed their personal engagement with school events, teachers, and administration. Both FGDs and caregiver interviews invited participants to explain their views of mental health. FGD facilitators and interviewers were trained on trauma triggers to ensure a safe space for participants.

All research protocols were approved by the Institutional Review Board (IRB) at Washington University in St. Louis (IRB ID# 201905151), the Director of the charter school system, and principals at all participating schools.

### Data Analysis

The survey sample comprised two study groups: (i) the first group consisted of students born outside the US, and (ii) the second included students born in the U.S. but whose parents or grandparents were born abroad. Sample estimates were calculated assuming statistical power of 80% and a two-sided alpha of 0.05 to detect a difference between the two study groups at an effect size of *d* = 0.40. Calculations resulted in a required sample size of 98 students in each group, for a total of 196 students. Demographic data were first summarized for the full sample and each of the two comparison groups. *T*-tests were used to assess the statistical significance of differences in outcomes between the U.S.-born and immigrant sub-groups; chi-squared tests were used to assess differences in binary outcomes. To assess evidence for the immigrant paradox, Ordinary Least Squares regressions were used to estimate all outcomes of interest for students born outside the U.S., controlling for age at arrival in the U.S., gender, years in the U.S. and an interaction term between gender and years in the U.S. The interaction term was included to examine whether years in the U.S. has a differential effect on well-being by gender. All quantitative analyses were conducted using the statistical software program, Stata14.

Prior to qualitative data analysis, a small team transcribed all audio-recorded interviews. The research team analyzed the data using thematic analysis and constant comparative methods (21). A team of research assistants with experience in social work, public health, refugee resettlement, and international development applied qualitative codes to each transcript using Dedoose, after it was ensured that inter-coder reliability was at least 66.7% (please see Table 2 for a summary of the codebook). Analysis focused on thematic factors influencing mental health outcomes for both newcomer and first-generation students. The lead analyst also considered acculturative stressors, instances of hope and resilience, and protective/promotive supports for newcomers on arrival through the lens of intersectionality by carefully noting intergroup differences in participant experiences based on gender, ethnicity, religion, immigration status, socioeconomic status, and disability, among others (22).

**Table 2:** Primary thematic codes analyzed.

**Theme**	**Code**	**Theme**	**Code**
Support	• Extra-curricular activities • Language support • Academic and career support • SEL support • Community support • Parental support • Peer support • Coping mechanisms	Intercultural factors	• Acculturation strategies • Reactions to American life and culture • Perceptions of refugees • Comparison between Arab and non-Arab migrants • Negotiating personal identities
School-specific	• Adjustment to new school • Sense of school belonging • School cultural and trauma competence	Structural factors	• Gender system • Employment and household finances • Political influences
Life experiences and challenges	• Mental health issues • Language challenges • Bullying or fighting • Discrimination • Peer influences • Within-group tensions • Inter-group tensions	Relationships	• Parent-child relationship • Parent-community relationship • School-child relationship • Peer relationship

## Results

### Quantitative Results

The survey was completed by 176 students, with attendance records obtained for 146 students. Within the full survey sample, 91 students reported being born in the U.S. and 85 students were born abroad. The majority of foreign-born students were born in Yemen (*n* = 44), Syria (*n* = 10), Iraq (*n* = 6), Palestine (*n* = 4), and other countries in the MENA region, though in one school a small proportion was also from Bangladesh (*n* = 13).

Table 3 summarizes the basic demographics for all participants, and for the two comparison groups. The average age of participants was 15 years. Females and males comprised 46% and 53%, respectively, of the sample, and one student reported a non-binary gender. Compared to the U.S.-born group, students in the foreign-born group were significantly more likely to speak Arabic at home (71% v. 36%; *P* < 0.001) and live in homes with a greater number of people per bedroom (1.78 v. 1.55; *P* = 0.010). No other inter-group differences in demographic characteristics were observed.

**Table 3:** Basic demographics of sample.

	**Full sample**	**U.S.-born group**	**Foreign-born group**	***p*-value of chi-squared or *t*-test**
Age	15.31	15.19	15.45	0.112
Gender				0.622
Female	0.46	0.46	0.46	
Male	0.53	0.53	0.54	
Other	0.01	0.01	0.00	
Religion				0.240
Christian	0.01	0.02	0.00	
Muslim	0.98	0.97	1.00	
No religion	0.01	0.01	0.00	
Language spoken at home				<0.001***
Arabic	0.53	0.36	0.71	
Bengali	0.07	0.01	0.14	
English	0.34	0.59	0.08	
Other	0.51	0.03	0.07	
People per house	6.27	6.16	6.40	0.423
Bedrooms per house	4.06	4.22	3.89	0.136
People per bedroom	1.66	1.55	1.78	0.010**
Sample size (n)	176	91	85	

Responses are missing for one observation for “people per house”, three observations for “bedrooms per house”, and three observations for “people per bedrooms.”

Overall, the primary analysis did not reveal statistically significant differences (defined at *P* < 0.05) between the foreign-born and U.S.-born study groups for any of the psychosocial or attendance outcomes of interest (see Table 4). Students in the U.S.- and foreign-born groups scored an average of 4.54 and 4.47, respectively (*P* = 0.632), on the hope scale, which can assume a value from 1 to 6. Measures of resilience, pro-social behaviors, depression, anxiety, externalizing symptoms, and perceived belonging were also statistically similar between the two groups. Finally, while students in the foreign-born group were tardy for a marginally greater number of classes and absent for marginally fewer classes, as compared to their U.S.-born counterparts, these differences were not statistically significant by conventional standards.

**Table 4:** Psychosocial and attendance outcomes, by group.

	**Full sample**	**U.S.-born**	**Foreign-born group**	***p*-value of *t*-test**	**Sample size**
Children's hope scale	4.50	4.54	4.47	0.632	175
	[0.97]	[1.07]	[0.85]		
SDQ: Pro-social sub-scale	8.07	8.27	7.86	0.124	176
	[1.79]	[1.69]	[1.88]		
CYRM scale	30.65	30.95	30.33	0.241	174
	[3.44]	[3.64]	[3.19]		
Hopkins checklist: Depression sub-scale	1.77	1.76	1.78	0.846	168
	[0.62]	[0.63]	[0.61]		
Hopkins checklist: Anxiety sub-scale	1.68	1.71	1.65	0.531	170
	[0.57]	[0.63]	[0.49]		
Hopkins checklist: Externalizing sub-scale	1.25	1.22	1.28	0.138	170
	[0.26	[0.21]	[0.31]		
Stressful life events checklist	2.89	2.73	3.09	0.291	177
	[2.31]	[2.28]	[2.34]		
Multidimensional scale of perceived social support (MSPSS)	5.40	5.58	5.20	0.078	175
	[1.43]	[1.20]	[2.28]		
Psychological sense of school membership (PSSM) scale	65.18	66.68	63.52	0.089	174
	[12.12]	[12.64]	[11.61]		
Class days tardy	1.74	1.59	1.92	0.398	124
	[2.20]	[2.12]	[2.30]		
Class days absent	3.87	4.24	3.41	0.177	124
	[3.37]	[3.54]	[3.13]		

Next, regression analyses were used to explore the immigrant paradox for foreign-born students, as well as the extent to which this paradox manifests differently for males and females. As shown in Table 5, each additional year a male student had lived in the U.S. was found to be associated with a 0.148 standard deviation decrease (95% CI[−0.252, −0.044]; *P* = 0.007) in resilience. Foreign-born male students also reported a marginal decline in perceived school belonging the longer they had lived in the U.S.; with each additional year of living in the U.S. male students experienced a 0.116 standard deviation decline in their PSSM score (95% CI [−0.232,0.001]; *P* = 0.051). However, these same relationships were not observed for female students.

**Table 5:** Estimating psychosocial outcomes according to gender and years in the U.S.

	**Children's hope scale**	**SDQ: Pro-social sub-scale**	**CYRM scale**	**Depression sub-scale**	**Anxiety sub-scale**	**Externalizing sub-scale**	**Stressful life events checklist**	**MSPSS**	**PSSM**
Male	0.149	0.555	0.349	−0.353	−0.142	−0.468	0.277	−0.190	0.260
	[−0.550,0.848]	[−0.218,1.328]	[−0.342,1.041]	[−1.213,0.508]	[−0.905,0.622]	[−1.411,0.474]	[−0.513,1.067]	[−1.048,0.668]	[−0.492,1.012]
Years in US	0.052	−0.062	0.044	−0.095	0.041	0.010	−0.213*	0.015	0.137
	[−0.129,0.233]	[−0.263,0.140]	[−0.134,0.222]	[−0.313,0.122]	[−0.147,0.228]	[−0.227,0.246]	[−0.422,−0.003]	[−0.208,0.239]	[−0.063,0.337]
Male*Years in US	−0.094	−0.100	−0.148**	0.030	0.011	0.097	0.058	−0.094	−0.116^+^
	[−0.197,0.010]	[−0.215,0.014]	[−0.252,−0.044]	[−0.100,0.160]	[−0.098,0.120]	[−0.039,0.233]	[−0.061,0.176]	[−0.221,0.034]	[−0.232,0.001]
Age at arrival	0.022	−0.089	0.01	−0.105	0.018	−0.001	−0.128	−0.019	0.075
	[−0.149,0.193]	[−0.278,0.101]	[−0.160,0.180]	[−0.308,0.098]	[−0.161,0.196]	[−0.225,0.223]	[−0.325,0.069]	[−0.231,0.193]	[−0.109,0.260]

Separate OLS regressions were used to estimate each outcome of interest. Outcomes were standardized; beta coefficients reflect the change in the outcome's standard deviation based on a one-unit change of the independent variable. Regressions estimating academic records are not included due to small sample size. Beta coefficients are statistically significant at ^+^p < 0.1, *p < 0.05, and **p < 0.01.

### Qualitative Results

The qualitative findings revealed several overarching themes that help explicate why newcomer students may fare similarly, on average, to students born in the U.S. First, shared Arab cultural heritage within schools and communities allowed newcomer students to access relevant language and mental health and psychosocial support. Second, peer relationships between newcomers and U.S.-born students seemed to strengthen both groups' language skills and formation of their ethnic identities. Findings also revealed shifting gender expectations and role hierarchies in newcomer students' relationships with parents and educators over time, possibly explaining the above statistical association between time spent in the U.S. and reductions in resilience among boys. The following sections examine these themes in greater depth.

#### Shared Cultural Heritage and School Network Allows for Relevant Service Provision

The DMA region's unique context as a multi-generation Arab enclave was a conspicuous theme across interviews. Many participants emphasized that “*most of the teachers…are also from the same background as us*” [Male-FGD, age 18] and were particularly attuned to the needs of both MENA newcomer and U.S.-born students, compared with general U.S. public schools. A guidance counselor who had worked in one of the schools for many years remarked that “*even if* [teachers] *don*'*t understand their students, they*'*ll find methods*” to provide them resources and support, making these schools “*one of the best spots for* [students] *to come into*.” She added that “*predominantly our admin here is Arab American, so we*'*ve got the language, so that barrier*'*s taken away… the culture, tradition*,” allowing newcomer students to “*feel like okay, a piece of home is here*.”

##### Beliefs, Praditions, and Practices

Many participants highlighted how the schools intentionally wove elements of Arab culture—belief systems, traditions, and practices of the MENA region—into the school culture. While a majority of students in the school system were of Arab descent, participants celebrated the diversity of student backgrounds and subcultures. A school official stated:

*The vast majority are Arabs. More than eighty percent. However, within the Arab culture, there are a million different cultures. There is diversity within the diversity. So, I*'*m not saying every teacher should be an expert in any culture. They should establish the culture in the school that we want to learn about each other. As long as I am aware, I*'*m trying to be aware, I*'*m trying to learn. I respect differences, and I accept differences*.

Another school official who was himself a refugee explained that newcomer students could “*see people that speak the same language. People that, even though this is not a religious school, have the same religious beliefs.”* Religious beliefs, he advanced, “*are so prominent in our culture that it basically sets our norms,”* bringing newcomer students comfort amidst resettlement chaos. A Jordanian-born student appreciated how, during the month of Ramadan, teachers adapted to the needs of fasting Muslim students. Teachers “*would all cut back on the homework, give us easy assignments, Arab or not Arab, they would all understand”* [M-FGD, age 18].

One student said parents chose these schools for their children “*because it's mostly Arabs, so they think it's a better influence on them*” [Female-FGD, age 15]. She felt that the school was “*basically the same thing* [as] *an American school, but you don*'*t feel like because you wear a scarf you*'*re* [an] *outsider or anything*.” An English teacher described how she deliberately affirmed students' religious practices, even during class: “*When that call to prayer comes, for many of our conservative students, that's huge in their family*.” Students could leave the class or move to the back of the classroom to pray, ensuring “*they know that that's not going to be penalized or that's not gonna be treated with disrespect*.”

The charter network provides annual cultural competency training for staff, which is especially tailored toward incoming non-Arab educators. A guidance counselor said the training oriented new staff to “*the culture, the tradition, the do*'*s and don*'*ts*” and takes place during the first week of school in order to “[give] *the staff resources* [so] *they know what to do*.”

Nevertheless, a few students reported perceived discrimination from classroom teachers, usually in the form of microaggressions. A group of U.S.-born girls in one FGD agreed that teachers should have more patience with newcomer students. One student clarified that teachers who “*have to be with* [newcomer students] *every single day constantly…get bothered by them*” [F-FGD, age 14]. She stated that “*they would throw a book at them and be like, ‘do you know how to write?' And if they say no*, [teachers] *would get really mad*.” Another student said that a substitute teacher told students to “*go back*” to their countries [F-FGD, age 14]. The student said the class became “*chaotic*” until “*the principal came in, and then we didn*'*t see the sub again*.” The administration's quick reaction to this discrimination reflected their intentional support for the diverse beliefs, practices, and languages of the MENA region.

##### Social and Emotional Learning Curriculum

Supports for newcomers' mental health and psychosocial well-being were provided in part by some schools' implementation of social and emotional learning (SEL) curricula. Key informants spoke about how newcomers faced accumulating acculturation stressors or tried to cope with stressful pre- or post-migration experiences. One teacher noted the challenge of teaching students whose relatives still live in war zones: "*Grandma is still in Yemen and the house was just bombed, now there's no place for Grandma to live… you can't teach English to a child who's worried about Grandma*.”

Schools implementing SEL curricula also acknowledged and appreciated its relevance to the broader student body. A school leader explained, “*it is very important not only for the student who is experiencing* [displacement and resettlement]*, but also very important for the student who had never those experiences before*.” He gave two reasons for this: (1) to encourage U.S.-born students to “*empathize with others*” and (2) to ensure “*they don*'*t take things for granted for themselves*.” A therapist for adolescents at a local community-based organization, who was also a refugee herself, similarly explained that newcomers “*from war-torn countries*” are “*automatically going to have more trauma*.” However, she recognized that “*students born here to people that were refugees* [are] *still carrying that trauma, like intergenerational trauma*.”

A guidance counselor at one school explained how SEL gives teachers tools to ask students “*what's going on, what*'*s bothering you*,” allowing students to express their range of feelings and emotions and to believe “*my teacher is here for me, mentally and emotionally first, where I'm at a 100 percent safe zone*,” so they can better focus on learning.

#### Peer Relationships in a Multi-Generation Arab Enclave

Students frequently perceived peer relationships to have a vital influence on their overall well-being. Asked how to improve supports for newcomer students, a student from Syria stated, “*I think friends sometimes play a bigger role than teachers and staff members*” [M-FGD, age 17]. As seen below, peer relationships were the primary mechanism through which newcomers accessed support and strengthened their identities within school settings.

##### Peer Relationships Between U.S.-Born and Newcomer Students

Peers identified the ability to use both Arabic and English as important for understanding students' differing identities and experiences. Multiple participants acknowledged that U.S.-born students benefit from exposure to peers with backgrounds different from their own. A school official noted how relationships between the two groups keep Arab tradition and culture alive for students with MENA heritage who have lived their whole lives in the U.S.:

*They're definitely helping in educating our kids as well. The more that we are here, we lose our culture, right? Or, we're creating our own, but we're losing the roots. Or, we start getting watered-down, almost. They help, you know, replenish that*.

Still, some students acknowledged tensions between groups. A student who arrived two years prior was bullied even “*after learning English*” because of having “*that accent*” [M-FGD, age 19]. When asked if bullying was an issue between U.S.-born and newcomer students, several U.S.-born girls described the behavior as “*more teasing*” [F-FGD, ages 17-18]. One girl discussed joking around with her other U.S.-born friends by pretending they had “*a boater accent*” [F-FGD, age 17]. However, she emphasized that “*if a person told us to stop, it*'*s not funny, then we would stop*.” A student who was born in Jordan mentioned that because he and many of his friends were “*all from the same place*,” they “*always make jokes about each other and about the culture*” without “*offending each other actually*” [M-FGD, age 18]. He elaborated that if “*people that aren*'*t from the same culture or the same background*” made a similar joke, he would not want to “*hang out*” with them.

Some students and teachers intervened when bullying or teasing occurred between peers, encouraging students to reflect on newcomer experiences and their parents' or their own experiences. One key informant described her approach when she overheard U.S.-born kids using the term “boater,” which was to ask if “*this person* [is] *less than you, when your family has only been here one year, and you're only this much more acculturated than he is*.” A student who was born in the U.S. but whose parents emigrated from Lebanon articulated the same awareness: “*the kid*'*s parents came here at a young age and they were probably teased. And you were born here and you*'*re teasing the other person that came exactly the way that your parents came”* [F-FGD, age 17].

Peer outreach and mentoring strategies were used to mitigate inter-group tensions. For example, U.S.-born students were asked to invite newcomers to play sports or to participate in other activities with them. One guidance counselor believed peer mentorship eased newcomer students' transition, while also allowing “*students from America* [to] *realize the blessings that they have*.” U.S.-born students also initiated this process themselves. One student described how she used Arabic as a vehicle to connect with “*friends from overseas*,” while also using friendship as a vehicle to maintain her Arabic language ability:

*Everybody in my family speaks English except my mom; I speak more Arabic with her. So, it helps me keep the Arabic language. The way I speak Arabic with my mom I speak with them. I have this friend, she*'*s from Syria, she*'*s new. So, I talk to her*. [F-FGD, age 17].

A student who arrived in the U.S. from Yemen two years prior illustrated how encouragement from majority U.S.-born peers increased his hope for his future when he won the election for student body president. He reported how he “[got] *up there and* [spoke] *full English”* which made him believe, “*if I dream about anything, I can do it*” [M-FGD, age 19]. Further, he felt that having “*a lot of friends' support to do that*” gave him the confidence to “*go to any university, to do everything in life*.”

##### Peer Relationships Between Only Newcomer Students

Participants also emphasized the importance of friendships among newcomers. One mother stated that her daughter's and son's friends were mostly refugees “*because they're the only ones that can connect with each other*.” A school-based guidance counselor also noticed how a “*common understanding of what they went through, that trauma”* led newcomer students to “*support one another.”* Participants noted that newcomer students often formed their first and strongest friendships in ESL class. One student said “*a lot of students talk in Arabic, and we talk* [to] *each other, then we go lunch together. That*'*s how we meet*” [F-FGD, age 17]. Similarly, a U.S.-born student said that four girls–from Saudi Arabia, Syria, and Lebanon–*“didn't know each other at first, but then they* [all] *got put into the ESL classroom. And they introduced themselves to each other. And now, three years, four years later, they*'*re still together”* [F-FGD, age 17].

Newcomer students also mentioned forming lasting friendships through extracurricular activities, such as robotics or soccer. A student who had been in the US for three and a half years “*didn*'*t know anybody*” upon arrival [M-FGD, age 18]. He soon discovered that the students he met via soccer “*were people just like* [him] *who came from overseas and into here, America*.” These friendships “*made us become more comfortable* [to] *expand out the group and start finding more people who were raised in war* [to] *become friends* [with] *as well*.”

##### Other Supports to Newcomer Well-Being

Participants described several factors specific to newcomer students that contributed to their comparable levels of well-being. One factor was the hope and joy of attaining a safe external environment. One mother stated that “*with the fear in Yemen, they were always very fearful and angry and always on edge*.” She explained that in the U.S., “*there*'*s no fear, they are happier. Here they are more relaxed, more comfortable*.”

Caregivers attributed their children doing well to the expansive, high-quality educational opportunities offered in the U.S. One mother who had lived in multiple MENA countries described the U.S. as a “*nice place, for education is very good. And especially for the kids, to have a bit of a future*.” Another mother said that U.S. schools “*teach the student to depend on himself and to build his personality, to be responsible*.” She explained that students in Jordan were given class assignments, then constantly reminded to complete them, whereas, students in the U.S. were given assignments, expected to take responsibility for completing them, and penalized with low grades if they did not. She also said that U.S. teachers will “*give you a chance* [to] *make it by* [giving] *other assignment*[s],” whereas “*in Jordan if you fail at doing this one—that's it*.”

Family safety and educational opportunities were perceived to promote well-being for all newcomer students; however, as discussed below, newcomer male students who had been in the U.S. for longer exhibited worse resilience than female students.

#### Shifting Gendered Social Scripts and Hierarchies

Both male and female newcomers grappled with societal norms in the U.S. not reflective of those in their countries of origin. These gendered effects of acculturation manifested in two primary relationships: (1) child-parent, and (2) student-school. The child-parent relationship revealed increased stressors for boys compared to girls. Additionally, girls were perceived as accessing support more effectively within the student-school relationship.

##### Gendered Expectations

Girls were often encouraged to pursue wider options for the future, such as higher education or health careers, and presented with more choices for self-expression than in countries of origin. In contrast, teenage boys' prospects seemed limited by adjusted expectations that they tone down physical contact with one another and accept women in authority roles. A female participant's father said:

*In Yemen, girls in general once they reach the age of fourteen, fifteen: they shy out; they wear the different styles; they don*'*t communicate with others; they start building their own inner circle that*'*s so private. And sometimes there could be a challenge. I think* [my daughter] *wouldn*'*t be the same person if she was in Yemen*.

Adolescent girls in the U.S. were also perceived to be more empowered to address unwanted attention from their male counterparts. A male student mentioned that his female peers do not tolerate sexist jokes; in fact, they “*get offended*” and “*will probably smack you*” [M-FGD, age 19].

A school official explained the changing norms for contact among boys, saying “*they're coming from a refugee camp. We're not big on physical contact here, right? No fighting, no, even horseplay…to some of them*, [fighting and horseplay is] *normal. It's what the guys do. Male roles versus female roles*.” One male student described his surprise in finding out that “horseplay” between boys could result in disciplinary action in the U.S.: “*The joke in Palestine is not like here. Here if you joke with somebody, you go to office. There we just joke, we hit each other, we slap each other, and they don't care*” [M-FGD, age 16].

##### Child-Parent Relationships

Caregivers indicated changes in relationships with their children in the U.S. as compared to their country of origin, with some of these dynamics transforming regardless of gender. This mostly seemed to be due to expectations that, as adolescents learn more English, they will assume greater responsibility acting as a family-community liaison. One mother stated, “*My daughter is really good with English, so if I needed something, I would tell her, and my daughter would go seek the help*.”

While one father said he would “*scream*” and “*argue*” with his children more in Yemen, he said that his relationship with them as adolescents in the U.S. is “*different, like there's no more parent saying, ‘Kids.' It's like we are in the same class*.” His wife said that she and her children have “*become like sister and brother, you know? We're friends more*.”

This changing caregiver-child relationship may have placed a greater comparative burden over time on adolescent boys who were expected to support their family financially while in school. A school-based ESL paraprofessional mentioned that “*some of ‘em* [boys] *come here a little older and they're also helping their families. They*'*re not just going to school, they also have full-time jobs*.” She explained that having to work negatively impacts boys' ability to study for tests and complete assignments “*because of the outside family issues, or stuff like that*.” A teacher acknowledged that these boys “*know that their life chances beyond school are limited because their role in the family is to be the primary breadwinner*.” The teacher also explained that adolescent girls did not have this same requirement.

The burden on adolescent boys was amplified when families were split up due to immigration processes. One father said, “*the visa process would go to part of the family, not to the whole family*.” For one family he knew, “*the father was delayed because of administrative process,”* which meant “*it was four years that the family* [had] *to live without the father, and he just came yesterday*.” According to one teacher, the elder son of a mother awaiting her husband said her son was “*now taking on some of the role of father in the family. So, he was with her to kind of deal with this younger brother*.”

##### Student-School Relationships

Newcomer students arrived with more rigid expectations that students should be deferential while instructors should be didactic. As one staff member said: “*The students that are refugees or come from another country have a higher respect to the teachers and the principal than the students that are here, ‘cause overseas it's top for them*.” Newcomer girls especially seemed to benefit from a shifting educator-student dynamic over time. They described relationships with teachers and staff at school in terms such as mentorship, which allowed them to develop advanced skills.

A classroom instructor who was of Arab descent but born in the U.S. said of two newcomer girls that she would “*literally do anything for them*.” She would “*talk to them about college and registering for college, stuff they need to do, community service*” and “*how* [to] *better your background and people like you and your community*.”

The FGDs indicated that girls received some school supports more than boys did. A male student pointed out that he knew a few peers who had gone to the guidance counselor, but “*specifically, the girls*” [M-FGD2, age 14]. A guidance counselor also noticed this imbalance, explaining how she felt that “*girls tend to understand this is better for them. Our boys feel like, again, it*'*s an Arabic mindset, unfortunately, that their ego, they can*'*t be special or singled out*.” Meanwhile, a male student perceived that within all-gender classrooms, teachers “*help* [girls] *more than the boys. Sometimes the boys, I ask them, they*'*re like, who cares*” [M-FGD, age 15].

A female student described her close relationship with a female teacher who “*had this damnit doll, where I*'*d beat the living crap out of it every day because I was so pissed off that whole year, but you know… We learn to control our anger*” [F-FGD, age 17]. In fact, a coordinator for newcomer families at a local CBO further highlighted the protective—and intentional—nature of providing access to female service providers for adolescent girls, explaining that “*to deal with male, she is not going to be open up that much. She will be scared of judging or misleading, so we preferred, just with this population, to have female therapists and female case managers*.” Collectively, these supports for newcomer female students may have heightened their sense of well-being compared to their male peers.

Newcomer boys did seem to benefit from the less authoritarian educator-student relationship. A few boys described regarding teachers and staff more as friends than as formal mentors. One student proclaimed his teacher to be “*a teacher when he wanna be a teacher and your friend all day long*” [M-FGD, age 16]. Another participant described an administrator as someone whose office “*you can walk into and talk about whatever you want*,” mentioning he viewed him as “*an older brother who wants the best for you*” [M-FGD, age 17]. That this relationship had a negative side showed when one teacher said that some students “*realize that teachers cannot put a hand on* [them],” which “*gives them confidence and more gusto to act out*.”

## Discussion

Recognizing the myriad stressful life events, interruptions to social support, and potential difficulties in adapting faced by immigrants, and refugees in particular, one is led to anticipate that foreign-born students may fare worse than second- or third-generation students born in the U.S. However, theories, such as the immigrant paradox, hypothesize that as immigrant families and generations adapt to American culture and norms, they tend to do worse off with respect to education and psychosocial outcomes (23). Under this assumption, one might instead expect refugee and immigrant students to exhibit improved mental health and psychosocial outcomes compared to their second- and third-generation peers. This study explores these two hypotheses by examining well-being among students born in the MENA region and U.S.-born students of Arab origin in one of the largest Arab enclaves in the U.S. Overall, our study found no statistically significant differences in key outcomes of well-being between the two groups; students from the MENA region and students whose parents originated from the MENA region exhibited similar levels of resilience, hope, and perceived school belonging, as well as comparable symptoms of depression, anxiety, and externalizing behaviors.

Qualitative findings help elucidate why foreign-born students seem to fare similarly to their U.S.-born peers. Insights from the qualitative data highlight two main themes: a shared Arab heritage and strong social networks among U.S.- and foreign-born adolescents in the DMA enabled newcomer students to seek culturally appropriate mental health and language supports, and the diverse peer relationships held by foreign-born students served as protective networks for these newcomers. Peer relationships included both those between newcomers as well as with U.S.-born students, which offered protective supports for both groups. While previous research has emphasized the protective effect of friendships with other first-generation adolescents, in particular (24), for foreign-born students with respect to health behaviors and outcomes, our findings suggest friendships with second- and third-generation immigrants may also help to support newcomers.

Qualitative findings also help explain the lack of evidence supporting the immigrant paradox in this setting. Results suggest that the high density of Arab individuals in the broader community and in the charter school system has a protective effect on U.S.-born students of Arab origin. Further, findings indicate that the peer interactions and friendships that develop between U.S.-born and newcomer students help to anchor U.S.-born students to their parents' heritage, language, and experiences before coming to the U.S., further strengthening intra-family relationships. Previous studies have found that second-generation adolescents who use both English and their parents' first language, as opposed to English only, exhibit fewer externalizing symptoms (25, 26). Students in the present study noted that the requirement of Arabic classes at school, along with the opportunity to practice Arabic with newcomer students, resulted in crossover benefits for their parental relationships.

The present study also finds evidence that the longer a foreign-born adolescent male has lived in the U.S., the worse off he is with respect to resilience. Previous studies have revealed similar trends, whereby immigrant students are more likely to exhibit externalizing symptoms the longer they have lived in the U.S. (27). Our qualitative analysis revealed how gendered social scripts and hierarchies from students' home countries begin to transform when foreign-born students immigrate to the U.S.; while these changing expectations may not affect students immediately, they appear to have an accumulative negative impact on students' well-being over time. For example, while navigating adjustments to the U.S., the changing parent-child relationship seems to shift greater responsibility to the child for boys, in particular. At the same time, female students appear to thrive from their relationships with teachers in ways that boys do not. Importantly, previous research demonstrates that supportive relationships can mediate the association between time in the U.S. and well-being outcomes, suggesting that girls' ability to leverage these teacher relationships in supportive ways may help to mitigate the risk of lowered resilience with increasing time in the U.S. (28).

Findings from this study should be considered alongside a few limitations. First, the final sample size for the survey was marginally smaller than deemed sufficient by sample size calculations. As such, it is possible that there are in fact differences between the two groups that would have been detected with a larger sample. Second, as all outcomes of interest were constructed from self-reported items, it is possible that responses were influenced by social desirability bias. However, all surveys were self-administered, and no identifying information was entered into the survey, increasing confidence in the validity of responses. Third, it is important to acknowledge who is able to resettle in the U.S., with admittance determined by politically driven nationality quotas (or bans) and strict eligibility requirements (29, 30). Often, government officials and processes favor refugees deemed most likely to contribute to the U.S.' economy and society (31). As such, many point to the “healthy migrant effect,” whereby healthier migrants self-select into immigration (or are more likely to be granted asylum, in the case of refugees) (32). Given that many of the foreign-born students in this study hold refugee status, it is possible that the aforementioned “healthy migrant effect” accounts for at least a portion of the parity observed between the U.S.- and foreign-born students with respect to mental health and psychosocial well-being. Lastly, it is important to recognize the limitations of the binary gender analysis presented in this study. While students were able to report a non-binary gender in the survey, only one student opted for this response, making the sample of such students too small to include in stratified gender analysis. Future research should consider how all gender identities interact with acculturation processes and pathways to well-being for foreign-born students.

## Conclusion

Although this study took place in a particularly large Arab enclave, it points to broader lessons that may be relevant to other settings. For example, schools and local organizations that service newcomer families may usefully consider ways to foster friendships and peer-based mentorships between foreign-born students from different countries as well as between U.S.-born and foreign-born students. Facilitating meaningful interaction between foreign-born students and second-generation students who speak the same non-English language at home should also be prioritized, whether through peer “buddy systems” or language clubs. Further, thoughtful framing to students around the mutual benefits of such friendships should be emphasized in order to engender reciprocal acculturation and optimize supports for both groups (33). Such lessons and dialogues can be integrated into existing SEL curricula for students.

Efforts should also be made to support teachers' capacity for serving as informal mentors to foreign-born students, as was shown to facilitate resilience among newcomer girls in the present study. Relevant trainings for teachers and other service providers, which might usefully include content on SEL and resilience, might also review typical gendered expectations in adolescents' home countries and encourage dialogue on how these expectations may shift as they navigate their old and new cultures. This study highlighted the usefulness of providing a yearly cultural competency training for school staff; schools, mental health service providers, and community-based youth organizations in other contexts should also administer similar cultural competency trainings on a regular basis to ensure newcomer students receive culturally appropriate support.

This study adds to the limited literature on foreign-born students' psychosocial well-being and available supports systems in an ethnic enclave comprised of individuals with shared cultural heritage. Findings from this study highlight protective factors at the peer, school, and community levels that work to support both foreign-born and U.S.-born second- and third-generation students in this context. While some of these protective factors may arise naturally within the environment of an Arab enclave, several lessons learned can nonetheless be usefully adapted to other contexts in the U.S.

## Data Availability Statement

The datasets presented in this article are not readily available because the datasets generated and/or analyzed during the current study are not publicly available due to IRB restrictions but are available from the corresponding author on reasonable request. Requests to access the datasets should be directed to lindsaystark@wustl.edu.

## Ethics Statement

The studies involving human participants were reviewed and approved by All research protocols were approved by the Institutional Review Board (IRB) at Washington University in St. Louis (IRB ID# 201905151), the Director of the charter school system, and principals at all participating schools. Written informed consent to participate in this study was provided by the participants' legal guardian/next of kin.

## Author Contributions

LS and CA: study design. IS and MR: data collection. IS, CB, and AG: data analysis. IS and AG: original draft writing. All authors: review and editing. LS had full access to all of the data in the study and takes responsibility for the integrity of the data and the accuracy of the data analysis.

## Conflict of Interest

The authors declare that the research was conducted in the absence of any commercial or financial relationships that could be construed as a potential conflict of interest.
